# Innovative Powder Pre-Treatment Strategies for Enhancing Maraging Steel Performance

**DOI:** 10.3390/ma18020437

**Published:** 2025-01-18

**Authors:** Drahomír Dvorský, David Nečas, Esther de Prado, Jan Duchoň, Petr Svora, Ondřej Ekrt, Angelina Strakošová, Jiří Kubásek, Dalibor Vojtěch

**Affiliations:** 1 Institute of Physics, Czech Academy of Science, Na Slovance 1999/2, Praha 8, 182 21 Prague, Czech Republic; prado@fzu.cz (E.d.P.); duchon@fzu.cz (J.D.); svora@fzu.cz (P.S.); ekrt@fzu.cz (O.E.); strakosova@fzu.cz (A.S.); 2 Department of Metals and Corrosion Engineering, Faculty of Chemical Technology, University of Chemistry and Technology Prague, Technická 5, Praha 6—Dejvice, 166 28 Prague, Czech Republic; david.necas@vscht.cz (D.N.); jiri.kubasek@vscht.cz (J.K.); dalibor.vojtech@vscht.cz (D.V.); 3 University Centre for Energy Efficient Buildings, Czech Technical University in Prague, Trinecka 1024, 273 43 Bustehrad, Czech Republic

**Keywords:** powder metallurgy, heat treatment, mechanical properties, SPS

## Abstract

Maraging steel is a high-performance material valued for its exceptional properties, making it ideal for demanding applications such as aerospace, tooling, and automotive industries, where high strength, toughness, and precision are required. These steels can be prepared by powder metallurgy techniques, which offer new processing possibilities. This paper introduces novel thermal powder pre-treatment and its impact on the final mechanical properties. Solid solution pre-treatment results in a modest improvement in strength (from 972 MPa to 1000 MPa), while the use of pre-aged powder achieves the highest strength (1316 MPa) and lowest ductility (2.6%). A self-composite material is created by mixing pre-treated powders with the same chemical composition but different properties. Such material was characterized by intermediate strength (1174 MPa) and ductility (3.1%). Although challenges such a porosity and oxidation were present, this approach allows for tuning of mechanical properties by mixing pre-treated powders, offering significant potential for advanced engineering applications.

## 1. Introduction

Maraging steels are an exceptional class of materials known for their combination of high strength, excellent toughness, and superior weldability, which make them indispensable in demanding applications such as aerospace, tooling, and automotive industries [1,2]. These steels are characterized by very low carbon content (typically below 0.03 wt.%) and significant amounts of alloying elements, such as nickel, cobalt, molybdenum, and titanium. The remarkable mechanical properties of maraging steels arise from a martensitic microstructure and the precipitation of intermetallic phases, such as Ni_3_X (X = Mo, Ti) and Fe_2_Mo [3,4], during aging treatments.

Traditionally, maraging steels are produced via conventional melting and casting processes, followed by extensive thermomechanical treatments. However, the rise of advanced manufacturing methods, particularly powder metallurgy, has introduced new possibilities for material customization. Powder metallurgy techniques, including additive manufacturing and spark plasma sintering (SPS), enable precise control over microstructure and mechanical properties while reducing material waste and processing time [5,6,7].

Among these, SPS has garnered attention due to its ability to rapidly consolidate powders at high temperatures and pressures, resulting in fine-grained microstructures and near-net-shape components [6]. While additive manufacturing methods, such as selective laser melting, are convenient for fabricating complex geometries [8,9,10], sintering processes like SPS excel in processing simpler shapes with tailored properties and minimal defects [6,11]. Nevertheless, the SPS method remains underexplored, especially in the context of maraging steel [7,12,13].

A key advantage of SPS is the potential to integrate powder pre-treatment steps, such as solution treatment and aging, to manipulate microstructural evolution before compaction. This approach offers a unique pathway to tune the mechanical properties of sintered materials by leveraging the synergistic effects of powders with different pre-treatment histories. For instance, pre-treated powders allow the combination of particles with distinct properties into a composite-like material, where harder particles provide reinforcement, and softer, ductile particles act as a matrix [14].

This concept is commonly observed in composite materials; however, reinforcement and matrix are usually based on different materials (e.g., a combination of metal matrix and ceramic reinforcement). On the other hand, powder pre-treatment opens new avenues for optimizing the balance between strength and ductility in maraging steels, while preserving the chemical composition (metal matrix and metal reinforcement). Pre-treatment techniques have been employed to enhance the performance of various metallic systems. However, their application to maraging steels remains relatively unexplored.

Surface chemistry and oxidation during powder pre-treatment also play a critical role. Oxide layers, often formed during heat treatment, can act as barriers to diffusion or sites for stress concentration, affecting the mechanical integrity of sintered parts. While oxide removal methods are well established, their interplay with mechanical properties, especially in multi-phase systems, requires further investigation. Addressing these challenges is essential for unlocking the full potential of powder pre-treatment in maraging steel processing.

This paper introduces a novel approach to tailoring the mechanical properties of maraging steel through thermal powder pre-treatment, followed by SPS. By exploring the effects of solution-treated, aged, and mixed powders, we aim to elucidate the microstructural mechanisms governing property variation and highlight the potential of this method for broader applications in metallurgy. Our findings not only contribute to the understanding of maraging steel behavior but also lay the groundwork for future advancements in powder metallurgy and tailored material design.

## 2. Materials and Methods

### 2.1. Material Preparation

This study used commercially available powder of maraging steel with the following composition: 19.3 wt.% of Ni; 9.2 wt.% of Co; 5.1 wt.% of Mo; 0.7 wt.% of Ti; 0.1 wt.% of Al, Cr, Mn, and Si; and C content of less than 0.03 wt.%. This powder, which is usually used as a precursor for 3D printing. The oxygen content was measured using G8 Galileo by Bruker manufacturer (Karlsruhe, Germany). This method allows the determination of oxygen content at the sub-ppm level. The powder was placed into crucible made of Al_2_O_3_ and heat-treated at 820 °C for 1 h under Ar atmosphere. Powder was subsequently cooled down in water. The powder was filtered, rinsed with ethanol, and dried at 50 °C in a drier. Powder in this state is referred to as T4. The aging was performed at 490 °C for 6 h with slow cooling on air. The powder in this state is referred to as T6. The heat treatment steps were based on the previous studies [4,6]. The surface of the powder after heat treatments was covered with oxides. These oxides were removed using solution of 500 mL HCl, 500 mL H_2_O, and 3.5 g of urotropine. The powder was filtered and rinsed in ethanol after removal of oxides. It was then dried at 50 °C in a drier. Powders in T4 and T6 states were mixed together in the ratio of 1:1. All powders were sintered by spark plasma sintering (FCT System HP-D 10, Denkendorf, Germany) at temperature of 1100 °C for 10 min with pressure of 21 MPa. The conditions were based on previous studies; however, even though the applied force was the same in this paper, the pressure level was lower due to the larger diameter of the final product [4,6].

### 2.2. Microstructure Characterization

For microstructure characterization, the samples were ground on the SiC papers P80-P4000; afterward, they were polished on diamond pastes of 3 µm and 1 µm. The final polishing was performed on the Eposil F suspension. Samples were etched in the nital (10%) solution. Characterization of the samples was performed using TescanVEGA3 LMU scanning electron microscope (SEM) equipped (Brno, Czech Republic) with energy-dispersive spectroscopy (EDS) and light microscopy (OM) using Observer.D1m, Carl Zeiss AG (Oberkochen, Germany). The porosity was measured by image analysis of 10 pictures using ImageJ (version 1.52a; Madison, USA). X-ray diffraction (XRD) measurements were performed using PANalytical X’Pert Pro (PANalytical, Almelo, The Netherlands) in Bragg–Brentano geometry with a Co anode and an X’Celerator position-sensitive detector. The patterns were processed using X’Pert HighScore Plus software (version 5.2(5.2.0.31529); Malvern Panalytical B.V. Almelo, The Netherlands) with access to a PDF5 database to perform phase identification. Finally, Rietveld refinement was carried out using Topas V3 for quantitative phase analysis [15]. Thin TEM lamellae specimens were prepared by SEM/FIB lift-out technique on an FEI Quanta 3D FEG (Hillsboro, OR, USA). An observation of the microstructure was performed by Conventional Transmission Electron Microscopy (CTEM) and Scanning Transmission Electron Microscopy (STEM) with a High-Angle Annular Dark-Field (HAADF) detector using a FEI Tecnai G2 F20 X-TWIN microscope with a double-tilt specimen holder operated at 200 kV.

### 2.3. Mechanical Properties

Compressive and tensile properties were measured using INSTRON 1362 machine (Norwood, MA, USA) at room temperature at a strain rate of 0.001 s^−1^. Cylindrical samples with a diameter of 5 mm and 7.5 mm high were used for compressive tests. Compressive yield strength (CYS) and ultimate compressive strength (UCS) were determined from compressive curves. Tensile tests were performed on flat dog bone specimens with dimensions 10 × 1 × 3 mm in constrained area. Tensile yield strength (TYS), ultimate tensile strength (UTS), and elongation to fracture (A) were determined from compressive curves. Three measurements were performed for each material.

## 3. Results and Discussion

### 3.1. Microstructure

**Figure 1:**
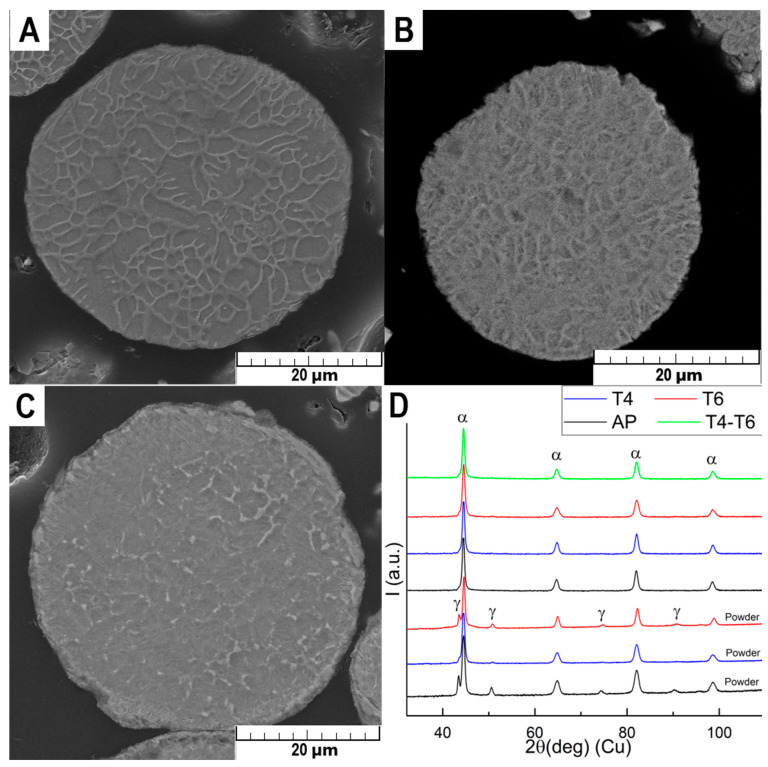
SEM micrographs of (**A**) atomized powder (AP), (**B**) solid solution-treated powder (T4), (**C**) aged powder (T6), (**D**) XRD patterns in logarithmic scale. The patterns have been vertically shifted for better visualization and converted to Cu radiation to facilitate comparison with the bibliography.

The atomized powder exhibited round-shaped particles ranging from 5 to 60 µm with a cell microstructure (Figure 1A) typical for atomized powders [16,17]. The present phases were identified by XRD as α martensite (92%) and retained γ austenite (8%), which were also observed by other authors [18]. Solid solution treatment led to the partial disruption of the cell microstructure (Figure 1B) and surface oxidation, increasing the powder’s oxygen content to approximately 1.4 wt.%. However, treatment with a deoxidating solution effectively reduced the oxygen content to 0.4 wt.%. The content of martensite increased to 99%. Upon aging, precipitation predominantly occurred inside the original cells and the content of martensite decreased to 89%, whereas austenite was located in the cell boundaries (Figure 1C).

**Figure 2:**
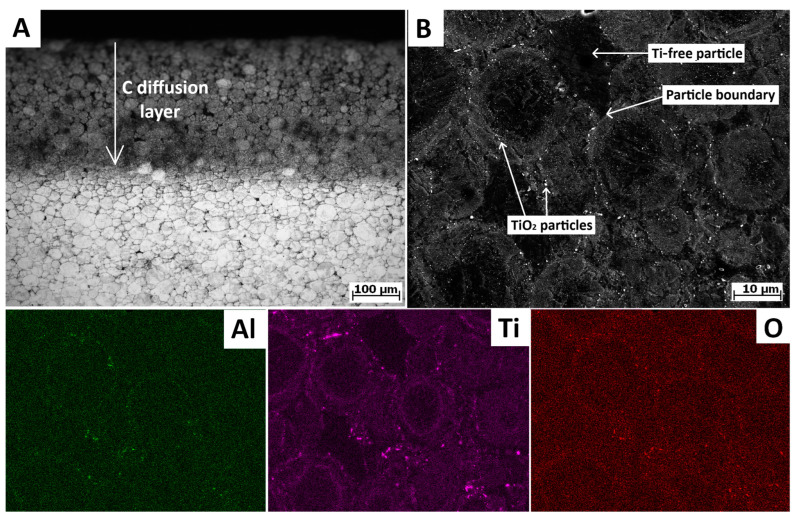
(**A**) Surface of sample after SPS showing layer of C penetration (optical microscopy), (**B**) SEM image and corresponding EDS analysis of T4 sample.

After sintering, carbon diffused from the carbon die into the material, reaching a penetration depth of approximately 250 µm (Figure 2A). This layer was subsequently removed by machining; thus, it did not interfere with XRD nor with the mechanical properties.

**Figure 3:**
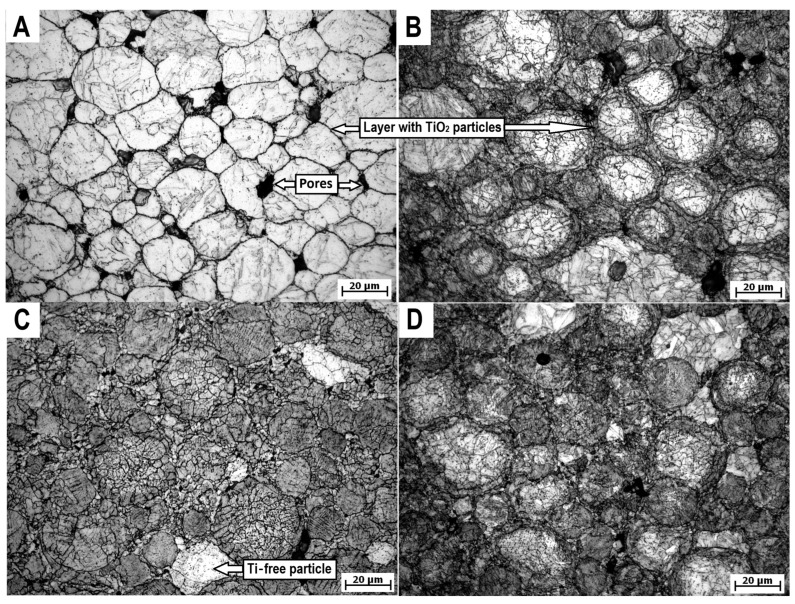
OM micrographs of compact samples from (**A**) atomized powder (AP), (**B**) solid solution-treated powder (T4), (**C**) aged powder (T6), (**D**) mix of T4 and T6 powder.

**Figure 4:**
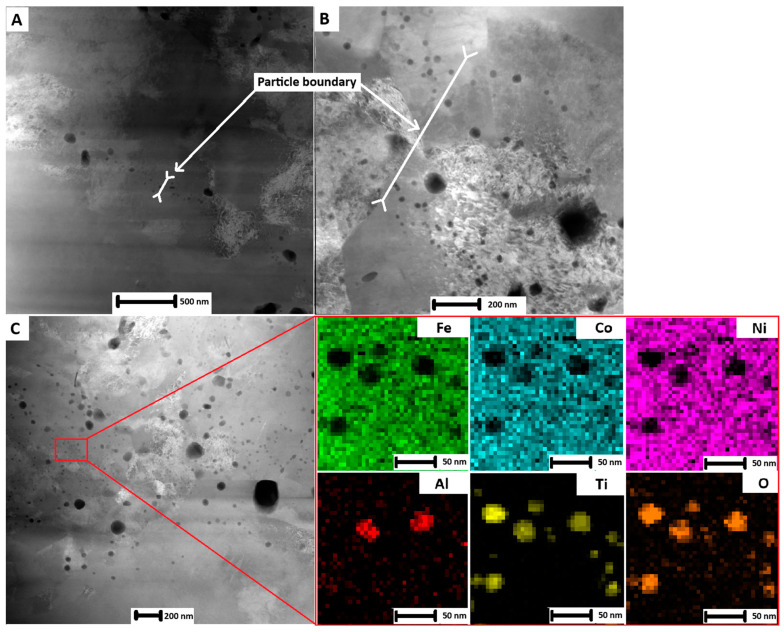
STEM images of (**A**) AP sample, (**B**) T4 sample, (**C**) T6 sample, and EDS analysis of oxides.

Inside the compact material, there was a porous structure (Figure 3A), likely due to the low sintering temperature and pressure (21 MPa). The measured porosity was approximately 3%, exceeding the ~1% reported by Strakosova et al. [6], presumably due to the larger sample diameter (30 mm). Menapace et al. [7] also observed some porosity after sintering milled powder at 1050 °C, despite using a higher pressure (60 MPa) [7]. These findings indicate that porosity depends highly on sintering temperature and also on the applied pressure. Each powder particle was enveloped by a thin oxide layer, comprising small round particles rich in Ti, Al, and O or N (Figure 2B and Figure 4A). The matrix in all cases consisted of 100% martensite (α), with a grain size of 0.5–10 µm. The sintering of the solution-treated powder yielded slightly lower porosity (~2%) (Figure 2B) probably due to the higher ohm resistance between particles. Few distinguishable particles (darker in Figure 2B or brighter in Figure 3C) were observed in all heat-treated samples. This was associated with oxidation of the powder during heat treatment, particularly at the upper layer of the powder pile in contact with gas, leading to the formation of TiO_2_ or Ti_2_N, thus depleting the solid solution. Ti_2_N was also observed by Zhu et al. [19] in 3D printed material. However, only a few particles were completely depleted of Ti, which is essential for age hardening. Thicker oxide layers surrounded each powder particle in the heat-treated samples (Figure 3B–D and Figure 4B,C), with an identical composition but a higher density of oxide particles and slightly larger size. The pre-aged sample exhibited less evident oxide layers but a more visible inner structure due to selective etching. The combined microstructure was achieved by mixing T4 and T6 powders, although a more distinguishable microstructure could be attained by combining AP and T6 powders.

Detailed images of the interparticle interface from the TEM observation of sintered samples are in Figure 4. There is an evident strip of oxide particles at the particle boundary. These particles likely originate from the oxide layer before sintering. During sintering this layer globularizes to form round oxide particles. This process is advantageous as the powder particles are connected by the metal matrix, therefore sustaining some ductility compared to the continuous oxide layer [20]. One can see that the sizes of oxide particles range from 5 to 150 nm, while the thickness of the oxide particle layer is up to 200 nm in the AP sample. The sample after heat treatment was characterized with the oxide particles with sizes ranging from 5 to 250 nm, while the thickness of the whole oxide particle layer was up to 2 µm. Therefore, the difference between AP and heat-treated samples was in the thickness of the layer and therefore also in the number of oxide particles. As these particles are TiO_2_ Ti_2_N and Ti-Al mixed oxides, they depleted the surrounding solid solution by alloying elements that are necessary for age hardening. Otherwise, the whole sample had a high concentration of dislocations. Interestingly, brighter particles that were depleted by Ti had a low concentration of dislocations. Unfortunately, no small phases of Ni_3_X (X = Mo, Ti) or Fe_2_Mo precipitates usually observed in maraging steels [3,4] were observed in any of the samples. This might be associated with a high sintering temperature (1100 °C), which is much higher compared to the temperature for solution treatment (820 °C). Therefore, even a short time of sintering resulted in the dissolution of these precipitates.

### 3.2. Mechanical Properties

**Figure 5:**
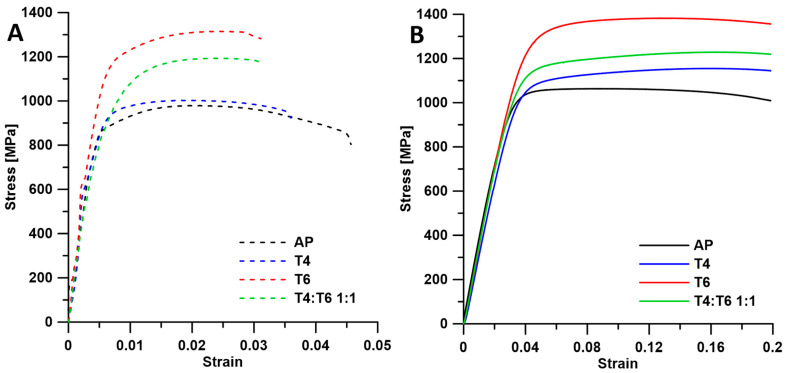
(**A**) Tensile (dashed line) and (**B**) compressive (solid line) curves of prepared materials.

Figure 5 summarizes representative compressive and tensile curves. The materials exhibited complete plasticity in compression without cracking. The UTS of the atomized powder was approximately 972 ± 8 MPa, which is comparable to the results obtained by SPS under similar conditions [5]. However, the lower elongation of about 4.5 ± 0.1% (compared with ~6% in [5]) could be attributed to the higher porosity (3% vs. 1%). The strength is also comparable with the 3D printed specimen [19]. However, elongation was also reduced in our case due to the presence of large and frequent pores. Solid solution treatment slightly increased the UTS to approximately 1000 ± 5 MPa but reduced elongation to 3.3 ± 0.3%, which is associated with the increased presence of oxides at the particle boundaries. Oxides at particle boundaries enhance strength but reduce ductility, as cracks propagate more readily along these interfaces [20]. Conversely, solution treatment slightly reduced UTS while improving elongation in the 3D printed sample [21]. The use of aged powder resulted in a more significant increase in UTS to approximately 1316 ± 13 MPa, with elongation decreasing to 2.6 ± 0.1%. The improvement of strength should be associated with the precipitation hardening; however, no fine precipitates were observed in TEM. Therefore, it seems that the strength is only improved by the presence of oxide particles. The greater amount of oxide particles at the particle boundaries also decreased elongation. The increase in strength was not as significant as that achieved by aging after SPS compacting (~1750 MPa) [5] or aging after 3D printing (~2160 MPa) [21], as precipitates probably dissolved during sintering despite the short duration at elevated temperature. However, the UTS reached is similar to the UTS after 1 h of aging in the 3D printed specimen [19]. Additionally, particles were partially or entirely depleted of Ti, thus hindering precipitation strengthening. Consequently, the inability of these particles to undergo precipitation strengthening results in lower strength compared to materials aged after sintering. The primary contributor to strength is the presence of oxides. However, by mixing T4 and T6 powders in equal ratios, a mid-range UTS of 1174 ± 12 MPa and an elongation of 3.1 ± 0.2% were achieved. Therefore, elongation is comparable with the T4 sample as the deformation may manifest in those particles, while strength is increased as harder T6 particles work as a reinforcement. Although further optimization is required, this observation suggests that mixing particles of the same material with different treatments can be used to tailor the desired mechanical properties while sustaining the same overall composition, which was the main goal of this study.

## 4. Conclusions

Heat pre-treatment of atomized powder offers a method to modify the mechanical properties of sintered products. However, achieving the same level of strength as post-heat treatment may be challenging due to the dissolution of precipitates during sintering. Nevertheless, the combination of harder and softer powders enables the tailoring of mechanical properties while maintaining the material composition. There are, however, several issues that require attention. The first is altering sintering conditions in order to achieve zero porosity. Increasing sintering temperature or pressure would result in lower porosity. The second is to avoid the depletion of the powder by Ti due to oxidation during heat treatment. This could be achieved by using a vacuum furnace or furnace with high-purity argon combined with consequent deoxidation. The third would be the optimization of heat treatment in order to obtain large overaged precipitates that would only partially dissolve in the solid solution during sintering, thus obtaining the desired microstructure with fine precipitates. Altogether, this method of mixing materials with the same composition but different properties seems to be promising for future research even for other materials.

## Data Availability

The original contributions presented in this study are included in the article. Further inquiries can be directed to the corresponding author.
